# β-NGF Stimulates Steroidogenic Enzyme and *VEGFA* Gene Expression, and Progesterone Secretion via ERK 1/2 Pathway in Primary Culture of Llama Granulosa Cells

**DOI:** 10.3389/fvets.2020.586265

**Published:** 2020-10-23

**Authors:** Ximena Valderrama, Cesar Ulloa-Leal, Mauricio Erciario Silva, Jose Goicochea, Silvana Apichela, Martin Argañaraz, Luciana Sari, Luis Paiva, Vicente Francisco Ratto, Marcelo Hector Ratto

**Affiliations:** ^1^Instituto de Investigaciones Agropecuarias (INIA), Osorno, Chile; ^2^Institute of Animal Science, Faculty of Veterinary Sciences, Universidad Austral de Chile, Valdivia, Chile; ^3^Department of Veterinary Sciences and Public Health, Faculty of Natural Resources, Universidad Catolica de Temuco, Temuco, Chile; ^4^Department of Surgery and Reproductive Biotechnology, Faculty of Veterinary Medicine and Zootechnics, Universidad Nacional Hermilio Valdizán, Huánuco, Peru; ^5^Instituto Superior de Investigaciones Biológicas (INSIBIO), CONICET-UNT, Facultad de Bioquímica, Instituto de Biología “Dr. Francisco D. Barbieri,” Química y Farmacia, UNT, San Miguel de Tucumán, Argentina

**Keywords:** *HSD3B1*, *CYP11A1*, *CYP19A1*, MAPK, U0126, corpus luteum, follicle, *STAR*

## Abstract

The beta-nerve growth factor (β-NGF) from llama seminal plasma exerts ovulatory and luteotrophic effects following intramuscular or intrauterine infusion in llamas and alpacas. In this study, we investigate the *in vitro* effect of llama β-NGF on the expression of genes involved in angiogenesis and progesterone synthesis as well as progesterone release in preovulatory llama granulosa cells; we also determine whether these changes are mediated via the ERK1/2 signaling pathway. From adult female llamas, we collected granulosa cells from preovulatory follicles by transvaginal ultrasound-guided follicle aspiration; these cells were pooled and incubated. After 80% confluence, the cultured granulosa cells were treated with β-NGF, β-NGF plus the MAPK inhibitor U0126, or luteinizing hormone, and the abundance of angiogenic and steroidogenic enzyme mRNA transcripts were quantified after 10 and 20 h by RT-qPCR. We also quantified the progesterone concentration in the media after 48 h by radioimmunoassay. We found that application of β-NGF increases the abundance of mRNA transcripts of the vascular endothelial growth factor (*VEGFA*) and the steroidogenic enzymes cytochrome P450 side-chain cleavage (P450scc/*CYP11A1*), steroidogenic acute regulatory protein (*STAR*), and 3β-hydroxysteroid dehydrogenase (*HSD3B1*) at 10 and 20 h of treatment. Application of the MAPK inhibitor U0126 resulted in downregulation of the genes encoding these enzymes. β-NGF also enhanced progesterone synthesis, which was prevented by the prior application of the MAPK inhibitor U0126. Finally, western blot analysis confirmed that β-NGF activates the ERK1/2 signaling pathway. In conclusion, our results indicate that β-NGF exerts direct luteotropic effects on llama ovarian tissue via the ERK 1/2 pathway.

## Introduction

It is now well-established that the beta-nerve growth factor (β-NGF) present in the seminal plasma of llamas and alpacas is the essential signal inducing the luteinizing hormone (LH) surge and ovulation in these species (1). Purified llama β-NGF also has a significant luteotrophic effect after intramuscular or intrauterine infusion in llamas and alpacas (2, 3). It is reported that plasma progesterone (since day 8 from ovulation) and corpus luteum (CL) diameter at day 16 are greater in llamas given two doses of 1 mg of purified β-NGF than those given a single dose (4). The increase of the vascular area of the CL is positively correlated to progesterone production in llamas treated with β-NGF (4, 5). Interestingly, administration of β-NGF is also shown to improve luteal function in cows (6).

In llamas, the preovulatory LH surge induced by purified llama β-NGF is more sustained than that the observed after GnRH administration (7, 8), suggesting that the luteotrophic effect of β-NGF could be due to this prolonged LH secretion profile. In this sense, systemic administration of β-NGF increased CL vascularization and upregulated the expression of cytochrome P450 side-chain cleavage (P450scc encoded by *CYP11A1*) and steroidogenic acute regulatory protein (*STAR*) mRNA transcripts enhancing plasma progesterone concentrations during the early luteal phase in llamas (9). Also, systemic administration of purified llama β-NGF induced a rapid shift from estradiol to progesterone synthesis in the preovulatory follicle in llamas as evidenced by the *in vivo* increase of the progesterone/estradiol ratio in the follicular fluid and upregulation of genes related to progesterone production (10).

The presence of NGF and its high-affinity receptor trkA in granulosa, theca, and luteal cells of mammalian species (11–13) suggests that the luteotrophic effect of β-NGF may be exerted not only by the prolonged LH secretion pattern, but also directly by acting at the ovarian level. In a recent study, the addition of purified llama β-NGF upregulated the expression of *STAR* and vascular endothelial growth factor (*VEGFA*) transcripts in primary culture of llama granulosa cells (10).

Previous studies investigating the effect of NGF/trkA on steroidogenesis in the ovary have been conducted in granulosa or theca cells from cows (14) and humans (15), species classified as spontaneous ovulators, and so might not be the same than those of llamas. After copulation, female llamas have been shown to increase their plasmatic content of β-NGF concomitantly occurring with the LH discharge from the posterior pituitary (16), which hampers the discrimination of β-NGF and LH effects on follicle cells *in vivo*. The use of an *in vitro* system may serve to elucidate the potential local effects of these hormones on luteal function, and thus, the effects of β-NGF are not confounded by those of the endogenous LH release.

In this study, we investigate the effect of β-NGF on steroidogenic enzymes and *VEGFA* gene expression as well as progesterone secretion and test whether these effects are mediated via ERK1/2 signaling pathway *in vitro* using a primary culture of granulosa cells collected from llama preovulatory follicles.

## Materials and Methods

### Animals

Llamas were kept at the llama research farm of the Institute of Animal Science, Universidad Austral de Chile, Valdivia, Chile (39°38‘S−73°5‘W and 19 m above sea level) and were maintained in pens and had access to natural pasture supplemented with hay and water *ad libitum*.

Experimental procedures were reviewed and approved by the University Bioethical Committee and were performed in accordance with the Chilean Animal Protection Act (2009) and the university animal care protocols.

### Semen Collection and Protein Purification

Semen was collected from five mature male llamas, twice per week for 5 months before the start of the experiments. An artificial sheep vagina was used as previously described (2). Each ejaculate was diluted 1:1 (vol/vol) with phosphate-buffered saline (PBS; Gibco, Grand Island, NY, USA) and centrifuged for 30 min at 1,500 × g at room temperature. A pool of sperm-free seminal plasma was stored at −20°C. Purification of β-NGF was performed in a two-step procedure as previously described (17, 18). In brief, seminal plasma was loaded into a type 1 macro-prep ceramic hydroxylapatite column (1 × 10 cm, 40 μm; Bio-Rad Laboratories, Hercules, CA, USA) previously equilibrated with 10 mM sodium phosphate, pH 6.8, and a flow rate of 0.5 mL/min. An eluted fraction showing a major protein on SDS-PAGE was concentrated in PBS (pH 7.4) using a 5 kDa cutoff membrane filter device (Vivaspin; Sartorius, Göttingen, Germany) and subsequently loaded onto a gel filtration column (SEC, hi Prep 26/60 Sephacryl S-100; Amersham Laboratories, Piscataway, NJ, USA). The purification procedure was carried out at room temperature at a flow rate of 0.5 ml/min using fast protein liquid chromatography (Amersham Laboratories, Piscataway, NJ, USA). Elution was performed isocratically using PBS at pH 7.4. The bioactive fraction after gel filtration was identified using an *in vivo* llama ovulation bioassay (17).

### Granulosa Cell Collection

Non-pregnant, non-lactating female llamas (*n* = 48) ≥ 6 years of age and weighing 120–150 kg were used from April to June. Llamas were submitted to transvaginal ultrasound-guided follicle ablation of all ovarian follicles ≥ 5 mm using a 19-gauge needle attached to a 5 MHz convex-array transducer to synchronize follicular wave emergence as described previously (19). The llamas were examined daily by transrectal ultrasonography using a 7.5-MHz linear-array transducer (Aloka SSD-500; Aloka Co., Ltd., Tokyo, Japan) to detect the emergence of a new preovulatory follicle. When a growing follicle ≥ 8 mm in diameter was detected (17, 18, 20), llamas were submitted to transvaginal ultrasound-guided follicle aspiration using a 5.0-MHz convex-array ultrasound transducer coupled to a 19-gauge needle as described previously (19, 21, 22) to collect granulosa cells by flushing the preovulatory follicle.

### Primary Culture of llama Granulosa Cells

Primary culture of llama granulosa cells was performed as previously described (10). Chemicals and reagents were purchased from Sigma–Aldrich Co., St. Louis, MO, USA, unless otherwise stated. In brief, follicular fluid collected from an individual female was centrifuged at 400 × g for 10 min. The cell pellet was then resuspended in 2 ml of Ham's F-12/DMEM and centrifuged again at 1,200 × g for 45 min in a 3 ml 50% Percoll column to separate granulosa cells from erythrocytes and interstitial cells. Purified granulosa cells were collected from the top of the Percoll column and washed twice by centrifugation at 400 x g for 6 min. Because a low number of granulosa cells were collected from individual animals, it was necessary to pool three animals after Percoll purification to get one biological sample. Granulosa cells were plated into 24-well culture plates (at 1 × 10^5^ cells/well) in Ham's F-12/DMEM supplemented with 10% fetal bovine serum and antibiotics (penicillin/streptomycin and gentamicin) and incubated in an atmosphere of 95% air, 5% of CO_2_ at 38°C and high humidity for 48 h. After 80% of confluence, the medium was replaced with serum-free Ham's F-12/DMEM and cells were treated with (i) control PBS, (ii) DMSO plus 10 μM of the MAPK inhibitor U0126 (Cell Signaling Technologies, Beverly, MA, USA), (iii) 30 ng/ml of LH (Lutropin-V; Vétoquinol Canada Inc., Lavaltrie, QC, Canada), (iv) 50 ng/ml of purified llama β-NGF, and (v) purified llama β-NGF plus 10 μM the MAPK inhibitor U0126 (added 30 min prior to β-NGF and remained for the whole period of treatment).

Five biological samples were used in both gene expression and progesterone secretion experiments (*n* = 15 llamas in each experiment); for each time and treatment, each biological sample was plated in 4 wells (i.e., experimental replicates) and treated as indicated above. For gene expression, cells were treated for 10 or 20 h; this time course was based on a previous report (10), in which significant changes in mRNA expression of *STAR* and *VEGFA* in primary culture of granulosa cells treated with β-NGF were detected.

### RNA Isolation and Real-Time PCR (Q-PCR) Analysis

Total RNA was extracted from granulosa cells using Trizol (Invitrogen Life Technologies, Carlsbad, CA, USA) according to the manufacturer's recommendations. The purity of the samples was analyzed using the Nanodrop 1000 (Thermo Fisher Scientific, Inc., Wilmington, DE, USA). One microgram of total RNA was converted to complementary DNA (cDNA) using the kit AffinityScript Q-PCR cDNA Synthesis (Agilent Technologies, Inc., Santa Clara, CA, USA) and Oligo-dT as per the manufacturer's instructions.

Brilliant III Ultra-Fast SYBR® Q-PCR master mix (Agilent Technologies, Inc., Santa Clara, CA, USA) was used to detect and quantitate the transcripts. Samples were run in triplicate and amplified in a QuantStudio 3 RT-PCR (Applied Biosystems, Foster City, CA, USA) thermocycler using the following thermal cycle conditions: one cycle at 95°C for 3 min, 40 cycles of 95°C for 5 s, 60°C for 30 s, and 72°C for 1 min. The coefficient of variation between samples ranged from 0.03 to 0.1 depending on the treatment. Data were analyzed using the thermocycler-associated software. For each sample, cycle threshold values for the assayed transcripts were normalized for total input cDNA (10 ng) concentrations using cycle threshold values for transcripts for the “normalizer” housekeeping gene, large ribosomal protein (RPLP0; (23)). The normalized values in each experiment were compared to a “calibrator” sample to determine the relative increase in the amount of the transcript. Primers for PCR were designed using Primer Express Software (PE Biosystems, Foster City, CA, USA). Llama primers for steroidogenic enzymes, *VEGFA*, and *RPLP0* transcripts and their expected size of amplified products and sequences have already been tested and previously described (9, 10). The accession numbers of gen data are shown in Supplementary Table 1. Primer sets for transcript amplification were used at a final concentration of 250 nM each. Data was analyzed using the thermocycler-associated software. Additionally, following amplification, the melting curves for the products were generated to ensure that the product represents a homogenous species.

### Progesterone Secretion From Primary Culture of llama Granulosa Cells

Progesterone concentration was determined from the primary culture of llama granulosa cells as previously described (10). Briefly, the procedure for follicular synchronization and aspiration, granulosa cell collection, and treatments were similar to the described above. However, for progesterone secretion, granulosa cells were cultured for 48 h after confluence. After the incubation period, the media was collected and stored at −20°C until it was assayed for progesterone. Progesterone concentration was determined by a solid-phase radioimmunoassay kit (RIA-CT KIP1458; DIASource ImmunoAssays SA Louvain-la-Neuve, Belgium) as previously reported (10). The intra-assay coefficient of variation was 0.26–3.57%, the minimum detectable limit 0.05 ng/ml, coefficient of variation of internal standard <3.87%, the limit of quantification observed for the assay was 0.45 ng/ml.

### MAPK/ERK Activity in Primary Granulosa Cells Culture

From 6 biological samples (*n* = 18 llamas total), 4 experimental replicates per sample were used to determine the phosphorylation state of ERK1/2 after β-NGF treatment. In brief, llama granulosa cells were cultured as described above and treated with (i) control PBS, 50 ng/ml of β-NGF for (ii) 0, (iii) 5, (iv) 10, and (v) 15 min, β-NGF plus 10 μm of MAPK inhibitor U0126 for (vi) 5, (vii) 10, and (viii) 15 min. After the incubation period, cells (1 × 10^6^) were lysed in 75 μl of lysis buffer containing 150 mM NaCl, 50 mM Tris-HCl, 1% Triton X-100, 1 mM EDTA, 1 mM EGTA, 1 mM phenylmethylsulfonyl fluoride, 1 mm sodium orthovanadate, 10 mg/ml leupeptin, 1.8 mg/ml aprotinin, 2 mM sodium fluoride, 2 mM sodium pyrophosphate, and 1 M dithiothreitol. Ten micrograms of protein from each sample were size-fractionated by 12% SDS-PAGE and then transferred to nitrocellulose membrane (Bio-Rad Laboratories, Hercules, CA, USA). Nonspecific binding was blocked by incubating the membranes in PBS (pH 7.4) containing 2% non-fat dried milk and 0.2% Tween-20. Membranes were then incubated overnight at 4°C with anti-phospho-MAPK p44/42 (ERK1/2) or anti-p44/42 (ERK1/2) rabbit polyclonal antibodies (1:1000; Cell Signaling Technologies, Beverly, MA, USA), followed by incubation at room temperature for 2 h with a goat anti-rabbit antibody conjugated with horseradish peroxidase (1:10,000; Cell Signaling Technologies, Beverly, MA, USA). The same membranes used to detect phosphorylated ERKs were stripped and blotted against total ERKs. Proteins were detected using an enhanced chemiluminescent detection system (PerkinElmer Life Sciences, Wellesley, MA, USA). The films were scanned and analyzed with a gel doc automated digitizing system (G:BOX Chemi XRQ; Syngene, Cambridge, UK).

### Statistical Analysis

Data for gene, ERK protein expression, and progesterone concentration were analyzed by the PROC MIXED procedure, including the effect of treatment, time, replicate, and their interaction. The Dunnett test was used to compare gene expression of all treatments with the control groups. If significant differences were detected, means were compared among groups using Tukey's test. All data are reported as mean ± SEM. Analyses were performed using the Statistical Analysis System software package SAS Learning Edition, version 4.1 (SAS Institute, Inc., Cary, NC, USA).

## Results

**Figure 1:**
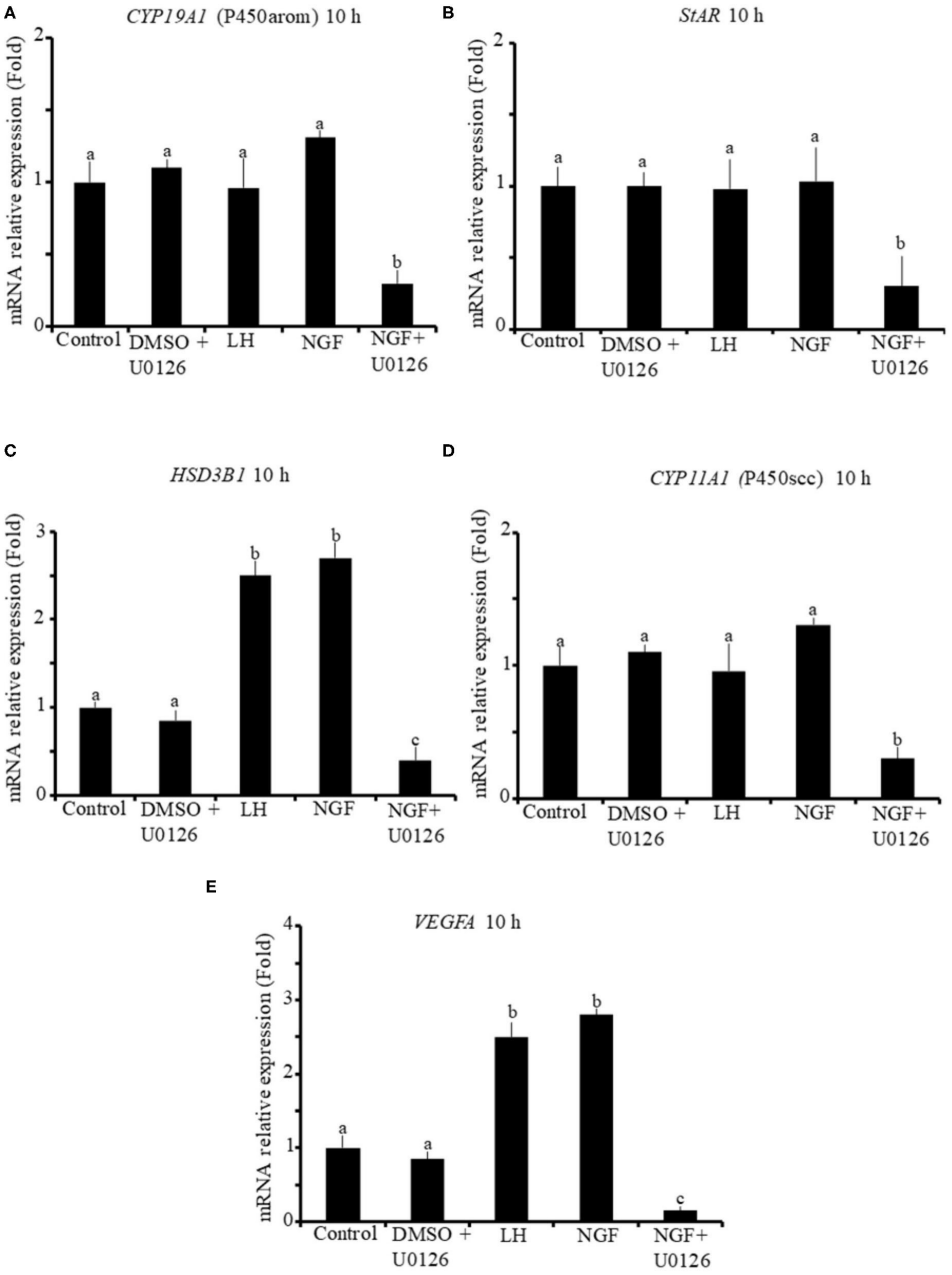
Relative mRNA abundance of **(A)** *CYP19A1* (P450arom), **(B)** *STAR*, **(C)** *HSD3B1*, **(D)** *CYP11A1* (P450scc) steroidogenic enzymes, and **(E)** *VEGFA* angiogenic factor in primary llama granulosa cell culture treated for 10 h. Mean + SEM; *n* = 5 biological samples; within each biological sample, 4 experimental replicates were performed; a, b, c superscripts indicate significant differences (*P* ≤ 0.01) between control and other groups.

**Figure 2:**
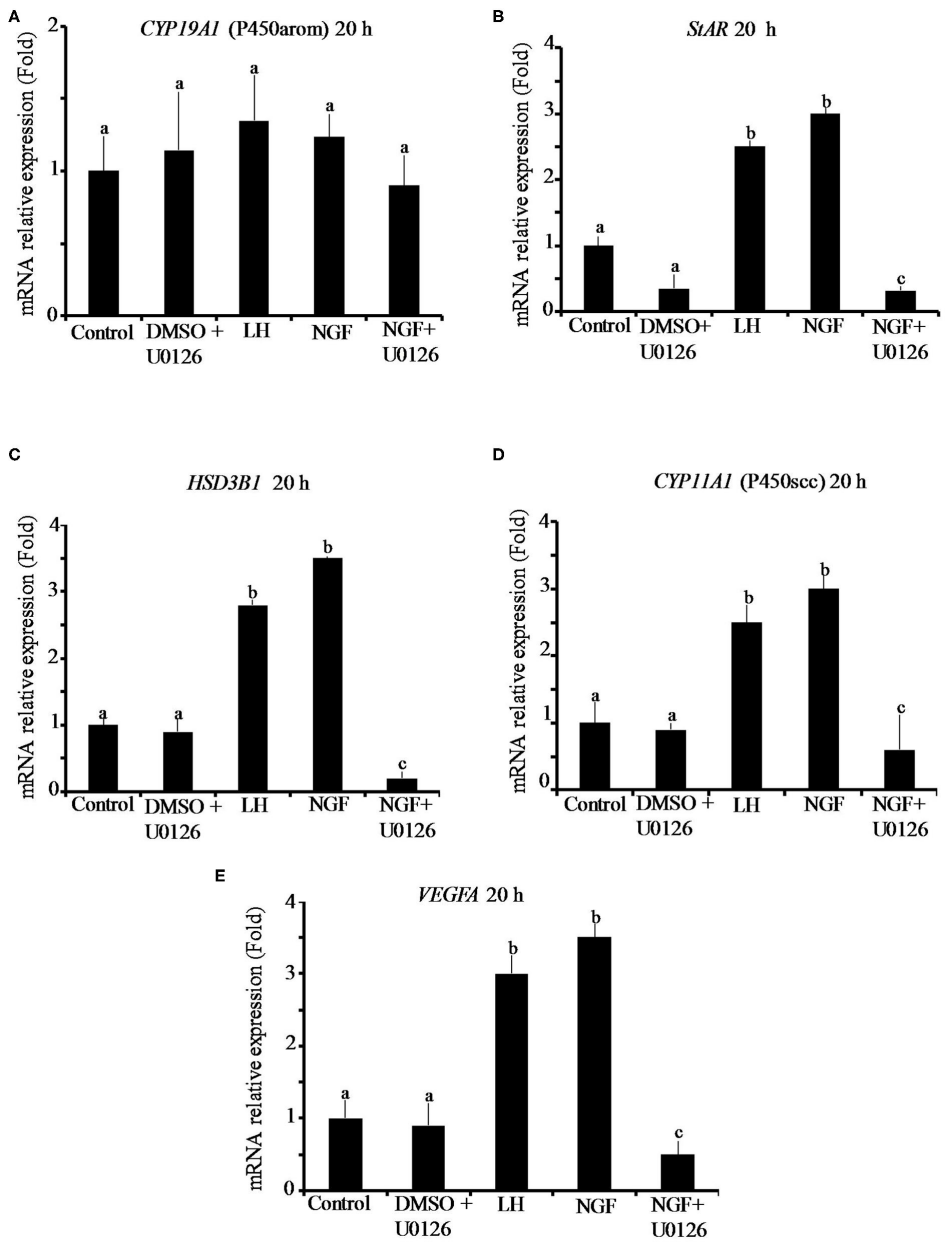
Relative mRNA abundance of **(A)** *CYP19A1* (P450arom), **(B)** *STAR*, **(C)** *HSD3B1*, **(D)** *CYP11A1* (P450scc) steroidogenic enzymes, and **(E)** *VEGFA* angiogenic factor in primary llama granulosa cell culture treated for 20 h. Mean + SEM; *n* = 5 biological samples; within each biological sample, 4 experimental replicates were performed; a, b, c superscripts indicate significant differences (*P* ≤ 0.01) between control and other groups.

The effect of β-NGF or LH treatment on mRNA expression of steroidogenic enzymes and *VEGFA* in llama granulosa cells are shown in Figures 1, 2. There was a significant (P ≤ 0.01) increase of 3 beta-hydroxysteroid dehydrogenase (*HSD3B1*) and *VEGFA* mRNA abundance in cells treated with β-NGF or LH after 10 h (Figures 1C,E). After 20 h, the mRNA abundance of *STAR* and *CYP11A1* (P450scc) steroidogenic enzymes also significantly increased; the *HSD3B1* and *VEGFA* mRNA transcripts remained upregulated (Figures 2B–E). As expected, neither β-NGF nor LH affected the mRNA expression of cytochrome P450 aromatase (P450arom encoded by *CYP19A1*) that is involved in estradiol synthesis (Figures 1A, 2A).

The addition of the MAPK inhibitor U0126 prior to the β-NGF treatment downregulated (P ≤ 0.01) the mRNA abundance of all steroidogenic enzymes and *VEGFA* transcripts after 10 h (Figures 1A–E). Most of the genes remained downregulated after 20 h, except *CYP19A1* (P450arom), which was not significantly different from control (Figures 2B–E). Cells treated with the U0126 inhibitor alone did not significantly change the mRNA expression of steroidogenic enzymes and *VEGFA* at any time.

**Figure 3:**
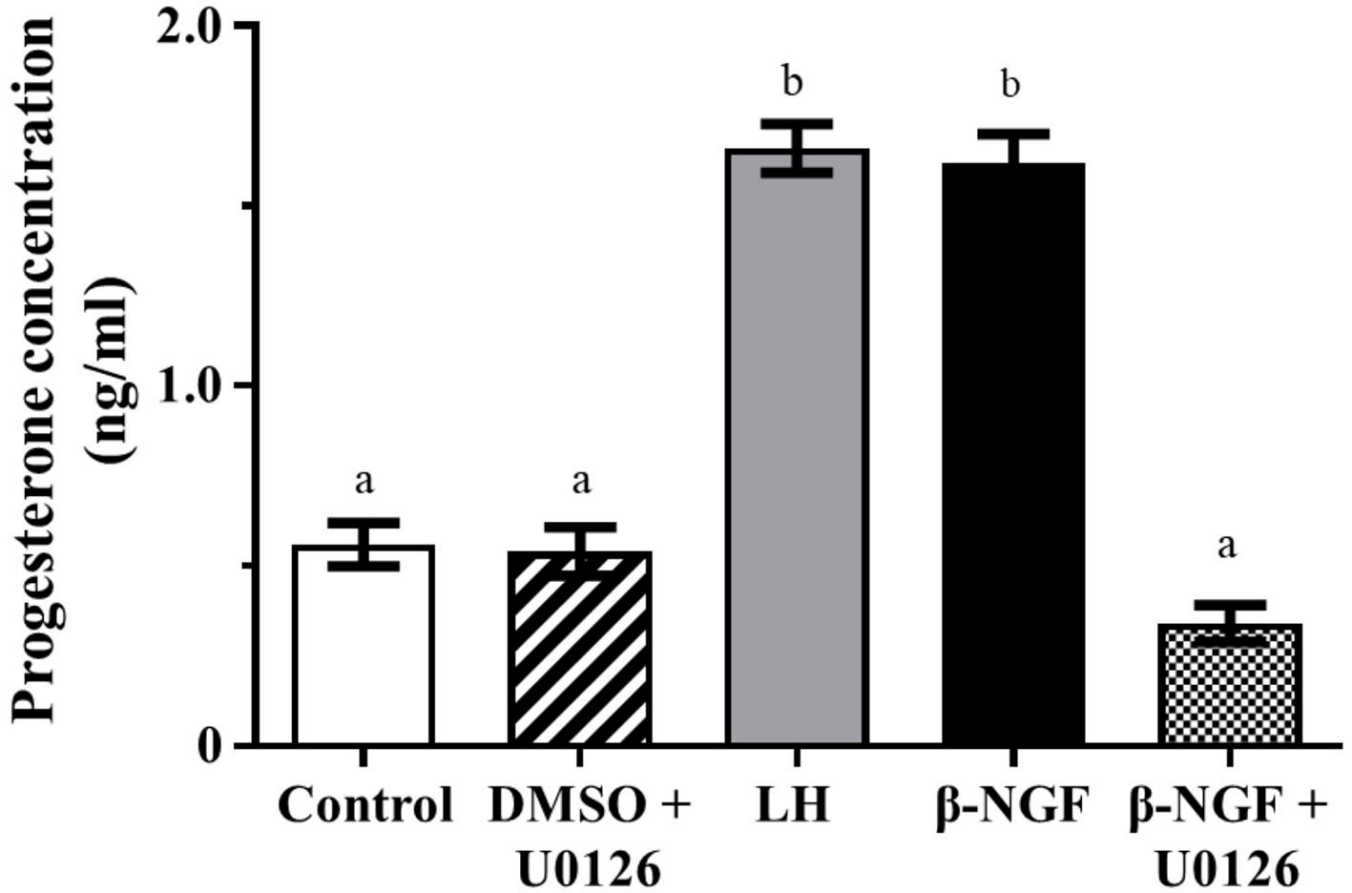
Progesterone secretion from primary culture of llama granulosa cells after 48 h. Mean ± SEM; *n* = 6 biological samples; within each biological sample, 4 experimental replicates were performed; a, b superscripts indicate significant differences (*P* ≤ 0.01) between control and other groups.

Progesterone secretion from llama granulosa cells was significantly (*P* ≤ 0.01) increased 48 h after β-NGF or LH treatment; the addition of the MAPK inhibitor U0126 prior to the β-NGF treatment prevented the increase of progesterone secretion as shown in Figure 3.

**Figure 4:**
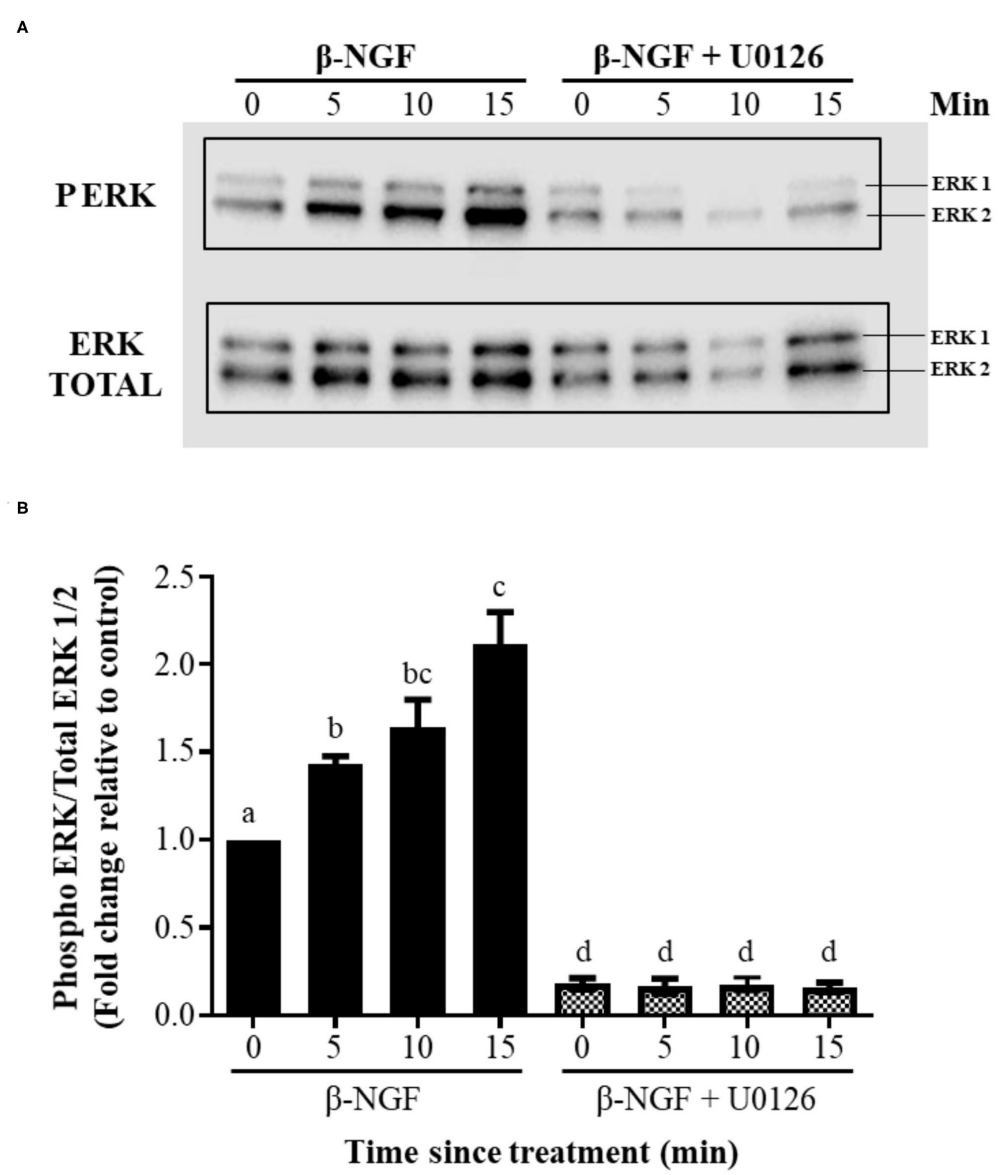
β-NGF induces phosphorylation of ERK1/2 in llama granulosa cells *in vitro*. **(A)** Western blot showing that NGF induces a rapid, time-dependent phosphorylation of ERK2, and to lesser extent, expression of ERK1 by the same time intervals. **(B)** Densitometric analysis of the blot shown in **(A)**, expressed as the ratio of phosphorylated ERK1/2 of total ERK. Values are mean + SEM; *n* = 6 biological replicates. a, b, c, d superscripts indicate significant differences (*P* ≤ 0.01) between control and the other groups.

Western blotting of llama granulosa cells treated with 50 ng/ml of β-NGF showed a significant (*P* ≤ 0.01), time-dependent increase of ERK2 phosphorylation after 5, 10, and 15 min of treatment; this increase exhibited a reduced intensity for ERK1. The addition of the inhibitor U0126 prior to β-NGF administration significantly (*P* ≤ 0.01) decreased the state of ERK1/2 phosphorylation (Figures 4A,B).

## Discussion

Neurotrophins, including β-NGF, are classically known for their role in neural growth and survival (24), but the relatively recent isolation and purification of β-NGF from the seminal plasma of llamas (17, 18) and also other species (25) has led to revisiting their roles in reproductive biology, emerging new ones (18, 26). Previous studies have linked NGF to ovarian development and innervation (27–29), but in the last years, evidence (1) indicates that β-NGF is involved in CL formation.

In this study, we investigated whether purified llama β-NGF exerts luteotropic effects on a primary culture of llama granulosa cells collected from preovulatory follicles. We show that llama granulosa cells treated with β-NGF upregulates steroidogenic enzymes and angiogenic transcripts after 10 and 20 h of treatment, and that this response is mediated by the ERK1/2 signaling pathway. Also, the β-NGF-induced increase of progesterone concentration is prevented by the MAPK inhibitor U0126, confirming that the biological actions of β-NGF are mediated, at least in part, by this pathway.

The first evidence of NGF receptors in the ovary was given by Dissen et al. (29), who detect low-affinity receptors in theca and also, to a lesser extent in granulosa cells of antral and preantral follicles of peripubertal rats. Subsequent studies confirm the expression of the high-affinity trkA NGF receptor in granulosa and theca cells of several species, including cows (14), rats (30), and humans (15). Binding of trkA receptors by its ligand β-NGF is known to elicit activation of the ERK1/2 signaling pathway (31, 32), including in granulosa cells (33). In line with this, our results show that the sole application of purified llama β-NGF to primary cultured llama granulosa cells results in activation of the ERK1/2 signaling pathway.

Expression of different steroidogenic enzymes is crucial for the ability of cells to synthesize steroids from cholesterol and its precursors. In the present study, we detect upregulation of *HSD3B1* mRNA transcript after 10 h of both β-NGF and LH treatments; and after 20 h, all the transcripts of the steroidogenic enzymes involved in progesterone secretion are also upregulated. Few studies address the effect of β-NGF on these enzymes; we have previously reported that β-NGF enhanced *STAR, CYP11A1*, and *HSD3B1* mRNA transcripts in llama granulosa cells *in vivo* (10), but these followed a distinct time expression pattern. Recently, Stewart et al. (6) report that the expression of steroidogenic enzymes in heifer luteal cells seems to be relatively similar to that reported here, resulting in increased circulating progesterone, albeit the expression of *HSD3B1*—which converts pregnenolone to progesterone—was unaffected.

In spontaneous ovulators, it can be expected that granulosa cells collected by follicle aspiration from a preovulatory follicle may exhibit some degree of luteinization due to the effect of the increasing LH release occurring at the end of the follicular phase. Conversely, in llamas, LH is released in response to the copulatory stimulus as this species is an induced ovulator. In line with this, Silva et al. (9) report that, in llamas treated with control PBS but not β-NGF or GnRH, plasma progesterone concentrations remain basal for up to 10 days. Although, in the present study, we did not evaluate the ratio of progesterone: estradiol, we previously reported (10) a marked ratio increase from 0.2 to 0.8 in control and β-NGF-treated animals, respectively, and this shift to progesterone production occurred after 20 but not 10 h following β-NGF treatment. Finally, the persistent detection of *CYP19A1* (P450arom catalyzing estradiol) reported in the present study also supports that the cells cultured here preserve their granulosa phenotype over the time analyzed, and thus, the effect of β-NGF on llama granulosa cells should be carefully interpreted as prosteroidogenic rather than luteinizing itself.

Time-dependent gene expression patterns of steroidogenic enzymes following LH have been detected in cultured bovine granulosa cells, resulting in enhanced expression of *CYP11A1* (P450scc) and *HSD3B1* transcripts; paradoxically, *in vivo* expression of these genes is reduced in granulosa cells collected after the LH surge when compared to those collected before (34, 35). The parallel gene expression profiles induced by β-NGF and LH reported here may be related to phosphorylation of ERK1/2, which also occurs following activation of LH receptors in preovulatory granulosa cells (36).

Interestingly, the application of the inhibitor U0126 alone did not affect gene expression, but its use combined with β-NGF downregulated virtually all steroidogenic and *VEGFA* transcripts in the llama granulosa cells. A plausible explanation could be that, once the EKR1/2 pathway was blocked, β-NGF exerts a paradox effect by activating (or causing predominance of) other transduction pathways that downregulate the constitutive expression of the genes analyzed here. For example, in some cell types, β-NGF-activating trkA receptors stimulate cell growth and survival, whereas the absence of trkA stimulates apoptosis via the low-affinity neurotrophin receptor, p75NTR (37). Expression of p75NTR is found in granulosa cells of humans (38) and squirrels (39); whether this is expressed in llama granulosa cells is unknown.

Consistent with our findings on intracellular pathways and gene expression, we also find that β-NGF application results in an ERK1/2-dependent progesterone synthesis in primary culture of llama granulosa cells. A previous study (40) reports that *in vitro* NGF microdialysis perfusion of cow luteal tissue but not in non-proliferative luteal cell culture, results in increased progesterone (and also local oxytocin) release from ovaries at early- and mid-luteal stage. This discrepancy between experimental conditions is likely to be related to cell culture settings as thecal cells plated in low but not high density also failed to secrete the steroids in response to NGF (14). Conversely to the evidence from llama and cows, in cultured human granulosa cells collected from preovulatory follicles, NGF application is shown to increase estradiol, whereas it decreases progesterone secretion (15). Perhaps there are species-specific variations on steroid output response to β-NGF in granulosa cells.

The CL is considered one of the most vascularized body structures; it receives the greatest rate of blood flow per unit of tissue compared to any organ of the body (41), and so angiogenesis plays an important role during the CL formation. The LH surge is considered a key signal to influence the expression of the VEGF that induces angiogenesis throughout the proliferation of preexistent endothelial cells (42). In human granulosa cells obtained from *in vitro* fertilization patients, NGF is shown to promote ovarian angiogenesis by enhancing the secretion of VEGF through the activation of the ERK1/2 pathway (43). Similarly, we found that, in llama granulosa cells, *VEGFA* mRNA expression rapidly increases after 10 h to remain relatively stable after 20 h of β-NGF and LH treatments, and this increase was dependent on the ERK1/2 pathway. Enhancement of *VEFG* mRNA expression is also known to occur by different signaling pathways in other cell types (44); whether β-NGF also activates other pathways in llama granulosa cells remains to be determined.

Previous studies show that, in llamas, CL develops after ovulation induced by seminal plasma (2) or purified β-NGF (7, 8, 18) and tended to be larger, regressed later, and produced twice as much progesterone than those resulting from GnRH treatments, supporting the notion that this effect might be related to the sustained LH release profile induced by β-NGF (7, 8, 17, 45). However, decreasing pharmacological doses of the GnRH analog, gonadorelin acetate, have been shown to affect the magnitude of the LH release in a dose-dependent fashion, but the consequent reduction in LH discharge had no effect on the CL diameter and plasma progesterone concentrations in llamas (46), indicating that the enhancement of luteal function induced by β-NGF occurs locally at the ovarian level rather than upstream on the classical LH mechanism.

In this *in vitro* study, both β-NGF and LH treatments equally increased genes involved in CL angiogenesis and steroid synthesis that resulted in a similar progesterone secretion, making it difficult to distinguish the contribution to the luteotropic effect of each hormone separately, and so it could be that, when acting β-NGF and LH together, there are synergic effects at the follicular level, similarly as shown to occur with LH and insulin administration in porcine granulosa cells *in vitro* (47, 48). In line with this notion, llamas in which ovulations were induced by β-NGF displayed enhanced expression of steroidogenic enzymes (9) and plasma progesterone concentrations (49) than those of ovulations stimulated by GnRH.

In summary, here we show and identify the intracellular pathway by which β-NGF exerts a direct luteotropic effect on ovarian tissue; however, further studies are required to determine whether synergic mechanisms exist that explain the β-NGF-related enhancement of luteal function and progesterone secretion reported *in vivo*.

## Data Availability Statement

The datasets presented in this study can be found in online repositories. The names of the repository/repositories and accession number(s) can be found in the article/Supplementary Material.

## Ethics Statement

The animal study was reviewed and approved by University Bioethical Committee (Universidad Austral de Chile) in accordance with the Chilean Animal Protection Act (2009) and the University animal care protocols, and the Animal Bioethical Committee of CONICYT (Chilean National Council of Science and Technology).

## Author Contributions

XV and LP were responsible for the development of the primary culture of llama granulosa cells and the execution of RT-QPCR and western blots experiments. CU-L, MS, JG, VR, and MR were responsible for granulosa cell collection in llamas using ultrasound-guided follicular aspiration and interpretation of gene expression and western blot data. XV, MR, SA, LS, and MA were responsible for the experimental design of the study and interpretation of gene expression and western blot data. MR and LP were major contributors in writing and editing of the manuscript. All authors participated actively in the analyses, interpretation of the data, read, and approved the final manuscript. All authors contributed to the article and approved the submitted version.

## Conflict of Interest

The authors declare that the research was conducted in the absence of any commercial or financial relationships that could be construed as a potential conflict of interest.
