# Supplementary material for: β-NGF Stimulates Steroidogenic Enzyme and VEGFA Gene Expression, and Progesterone Secretion via ERK 1/2 Pathway in Primary Culture of Llama Granulosa Cells
Source: Front Vet Sci. 2020 Oct 23;7:586265. doi: 10.3389/fvets.2020.586265 (PMC7645075; doi:10.3389/fvets.2020.586265)
Supplement: Supplementary file 1 [file Table_1.docx]

**Supplementary Table 1.** Identity and accession number of genes assessed in llama granulosa cells.

| **Gene** | **Identity** | **Gene ID** | **Gene Bank Accession N°** | **link** |
| --- | --- | --- | --- | --- |
| *CYP19A1* (p450Arom) | *C.ferus* aromatase (LOC102519005), mRNA | 102519005 | XM_006181316.2 | <https://www.ncbi.nlm.nih.gov/nuccore/XM_006181316.2> |
| *StAR* | *Vicugna pacos* steroidogenic acute regulatory protein (STAR), mRNA | 102533547 | XM_006201129.1 | <https://www.ncbi.nlm.nih.gov/nuccore/XM_006201129.1> |
| *HSD3B1* | *Vicugna pacos* 3 beta-hydroxysteroid dehydrogenase/Delta 5-->4-isomerase-like (LOC102528409), mRNA | 102528409 | XM_006216141.1 | <https://www.ncbi.nlm.nih.gov/nuccore/XM_006216141.1> |
| *CYP11A1* (P450scc) | *Vicugna pacos* cholesterol side-chain cleavage enzyme, mitochondrial-like (LOC102526586), mRNA | 102526586 | XM_006213724.1 | <https://www.ncbi.nlm.nih.gov/nuccore/XM_006213724.1> |
| *VEGF* | *Vicugna pacos* vascular endothelial growth factor A (VEGFA), transcript variant X8, mRNA | 102536403 | XM_015237403.1 | <https://www.ncbi.nlm.nih.gov/nuccore/XM_015237403.1?report=genbank> |
| *RPLP0* | *Bos taurus* ribosomal protein, large | 6175 | BT021080.1 | <https://www.ncbi.nlm.nih.gov/nuccore/BT021080.1> |
|  |  |  |  |  |
